# A study of gene expression markers for predictive significance for bevacizumab benefit in patients with metastatic colon cancer: a translational research study of the Hellenic Cooperative Oncology Group (HeCOG)

**DOI:** 10.1186/1471-2407-14-111

**Published:** 2014-02-20

**Authors:** George Pentheroudakis, Vassiliki Kotoula, Elena Fountzilas, George Kouvatseas, George Basdanis, Ioannis Xanthakis, Thomas Makatsoris, Elpida Charalambous, Demetris Papamichael, Epaminontas Samantas, Pavlos Papakostas, Dimitrios Bafaloukos, Evangelia Razis, Christos Christodoulou, Ioannis Varthalitis, Nicholas Pavlidis, George Fountzilas

**Affiliations:** 1 Department of Medical Oncology, Ioannina University Hospital, Ioannina, Greece; 2 Department of Pathology, Aristotle University of Thessaloniki School of Medicine, Thessaloniki, Greece; 3 Laboratory of Molecular Oncology, Hellenic Foundation for Cancer Research, Aristotle University of Thessaloniki School of Medicine, Thessaloniki, Greece; 4 Department of Medical Oncology, “Papageorgiou” Hospital, Aristotle University of Thessaloniki School of Medicine, Thessaloniki, Greece; 5 Health Data Specialists Ltd, Athens, Greece; 6 First Propaedeutic Department of Surgery, “AHEPA” Hospital, Aristotle University of Thessaloniki School of Medicine, Thessaloniki, Greece; 7 Division of Oncology, Department of Medicine, University Hospital, University of Patras Medical School, Patras, Greece; 8 Bank of Cyprus Oncology Center, Nicosia, Cyprus, Greece; 9 Third Department of Medical Oncology, “Agii Anargiri” Cancer Hospital, Athens, Greece; 10 Department of Medical Oncology, “Hippokration” Hospital, Athens, Greece; 11 First Department of Medical Oncology, “Metropolitan” Hospital, Piraeus, Greece; 12 Third Department of Medical Oncology, “Hygeia” Hospital, Athens, Greece; 13 Second Department of Medical Oncology, “Metropolitan” Hospital, Piraeus, Greece; 14 Oncology Department, General Hospital of Chania, Crete, Greece; 15 Department of Medical Oncology, Medical School, University of Ioannina, Niarxou Avenue, 45500 Ioannina, Greece

**Keywords:** Bevacizumab, Colon cancer, Gene expression, Predictive, Response rate, Survival, Biomarker

## Abstract

**Background:**

Bevacizumab, an antibody neutralizing Vascular Endothelial Growth Factor (VEGF), is licensed for the management of patients with advanced colon cancer. However, tumor biomarkers identifying the molecular tumor subsets most amenable to angiogenesis modulation are lacking.

**Methods:**

We profiled expession of 24526 genes by means of whole genome 24 K DASL (c-DNA-mediated, Annealing, Selection and Ligation) arrays, (Illumina, CA) in 16 bevacizumab-treated patients with advanced colon cancer (Test set). Genes with correlation to 8-month Progression-free status were studied by means of qPCR in two independent colon cancer cohorts: 49 patients treated with bevacizumab + chemotherapy (Bevacizumab qPCR set) and 72 patients treated with chemotherapy only (Control qPCR set). Endpoints were best tumor response before metastasectomy (ORR) and progression-free survival (PFS).

**Results:**

Five genes were significantly correlated to 8-month progression-free status in the Test set: overexpression of KLF12 and downregulation of AGR2, ALDH6A1, MCM5, TFF2. In the two independent datasets, irinotecan- or oxaliplatin-based chemotherapy was administered as first-line treatment and metastasectomies were subsequently applied in 8-14% of patients. No prognostically significant gene classifier encompassing all five genes could be validated in the Bevacizumab or Control qPCR sets. The complex gene expression profile of all-low tumor (ALDH6A1 + TFF2 + MCM5) was strongly associated with ORR in the Bevacizumab qPCR set (ORR 85.7%, p = 0.007), but not in the Control set (ORR 36.4%, p = 0.747). The Odds Ratio for response for the all-low tumor (ALDH6A1 + TFF2 + MCM5) profile versus any other ALDH6A1 + TFF2 + MCM5 profile was 15 (p = 0.018) in the Bevacizumab qPCR set but only 0.72 (p = 0.63) in the Control set. The tumor expression profile of (KLF12-high + TFF2-low) was significantly associated with PFS only in the Bevacizumab qPCR set: bevacizumab-treated patients with (KLF12-high + TFF2-low) tumors had superior PFS (median 14 months, 95% CI 2-21) compared to patients with any other (KLF12 + TFF2) expression profile (median PFS 7 months, 95% CI 5-10, p = 0.021). The Hazard Ratio for disease progression for (KLF12-high + TFF2-low) versus any other KLF12 + TFF2 expression profile was 2.92 (p = 0.03) in the Validation and 1.29 (p = 0.39) in the Control set.

**Conclusions:**

Our «three-stage» hypothesis-generating study failed to validate the prognostic significance of a five-gene classifier in mCRC patients. Exploratory analyses suggest two gene signatures that are potentially associated with bevazicumab benefit in patients with advanced colon cancer.

## Background

The high cost of targeted therapies as well as their conceptual definition as «targeting» specific molecular aberrations mandate the use of biomarkers in modern oncology practice [1]. Biomarkers are tumor and host characteristics that either define the natural course of a malignancy irrespective of therapy (prognostic) or the probability of patient benefit from a therapy administered (predictive) [2]. Although both are clinically relevant, less progress has been made in the field of the latter.

Angiogenesis is the process of new blood vessel formation and is pivotal for tumor growth, invasion and metastases [3]. Bevacizumab, a humanized monoclonal antibody that binds and neutralizes one of the main effectors of malignant angiogenesis, the Vascular Endothelial Growth Factor (VEGF), has been licensed for the treatment of patients with metastatic colorectal cancer combined with chemotherapy [4]. However, the rather modest improvement in response and survival outcomes achieved indicate the rich tumor heterogeneity and the probability that only a subset of tumors are amenable to VEGF modulation. The molecular characterization of tumors responsive to bevacizumab remains the Holy Grail for a worlwide community of investigators.

Genomic technologies are being widely used to study tumors at the molecular level. Since the extraction of RNA from formalin-fixed, paraffin-embedded (FFPE) tumor tissue has been optimized, microarray-based multigene expression profiling platforms have been developed for the identification of molecular signatures associated with various tumor characteristics [5]. The larger scale availabilty and more straightforward feasibility of performing quantitative PCR (qPCR) assays commonly led to attempts to adapt microarray signatures to qPCR methodologies.

In this study, we used a microarray platform to profile the expression of 24526 genes in a test set of 16 patients with metastatic colon cancer treated with bevacizumab, aiming to identify a select set of genes associated with superior outcome on bevacizumab. We then studied the expression of these genes using qPCR in an independent set of patients who received bevacizumab and in a control set of patients who were treated with chemotherapy only, in order to confirm their significance and to dissect their potential predictive from prognostic utility.

## Methods

Patients with chemonaive metastatic colon cancer who received first-line standardized chemotherapy protocols with or without bevacizumab between 2005 and 2009 in oncology centers affiliated with the Hellenic Cooperative Oncology Group (HeCOG), consented for the research use of their biologic material. FFPE blocks were fully annotated with clinicopathologic characteristics. The translational research protocol was approved by the Scientific Commitee, Papageorgiou Hospital, Thessaloniki, 185/8-10-2013. The patient sets consisted of three cohorts: a) the Test set (N = 16, patients treated with chemotherapy and bevacizumab) in which FFPE microarray analysis was performed in order to identify candidate genes predictive of bevacizumab benefit, b) the Bevacizumab qPCR set (N = 49, an independent cohort of patients treated with chemotherapy and bevacizumab) and the Control qPCR set (N = 72, patients treated with chemotherapy without bevacizumab). All patients had a performance status of 0-1. In the latter two independent sets, expression of the selected genes with possible predictive/prognostic significance was quantified by means of qPCR (Additional file 1: Figure S1 for REMARK diagram).

For RNA extraction from FFPE tumors, H&E sections were histologically reviewed and areas containing >50% tumor cells were marked; these were macrodissected from serial unstained sections at 8um after deparaffinization and submitted for RNA extraction with the RNeasy FFPE Kit (Qiagen, Hilden, D). For the Test Set, two series of RNA samples were prepared; one of these was submitted for Illumina profiling. RNA samples from the Test, Bevacizumab qPCR and the Control qPCR Sets were processed for reverse transcription and first strand cDNA synthesis with the Superscript III and random hexamers (Invitrogen/Life Technologies). All reagents and systems were used according to the instructions of the manufacturers. cDNAs were normalized at 25 ng/ul and stored at -20°C until use.

### Microarray methodology and analysis

We performed global gene expression profiling on the 16 patients of our test set using whole genome DASL (cDNA-mediated, Annealing, Selection, and Ligation) arrays (Illumina, CA), covering more than 24,000 transcripts. This technology overcomes the challenges of profiling partially degraded RNA, often extracted from FFPE samples and provides high-quality gene expression data [6]. We isolated 250 ng of total RNA in a concentration of 25 ng/μl, as required by Expression Analysis Inc (Durham, NC). The A260/A280 ratio of each RNA specimen exceeded 1.6. Outlier exclusion was based on the percent present call of the samples; detection rate >12000 transcripts. Microarray experiments were carried out at Expression Analysis Inc (Durham, NC) according to the manufacturer’s recommendations. The microarray data have been submitted to Gene Expression Omnibus as study GSE53127 and can be viewed at: http://www.ncbi.nlm.nih.gov/geo/query/acc.cgi?acc=GSE53127.

### 5-gene predictor validation with qPCR

The 5-gene predictor was evaluated on cDNA samples from the Bevacizumab qPCR and Control sets with qPCR and hydrolysis probes (TaqMan® MGB probes, Applied Biosystems/Life Technologies). The following premade assays were selected for amplicons matching the regions targeted by corresponding Illumina probes (assay ID, NM-reference, exon spanning, location, size): AGR2 Hs00356521_m1 (NM_006408.3, ex 7-8, 665, 69 bp); ALDH6A1 Hs00194421_m1 (NM_005589.2, ex 11-12, 1607, 131 bp); KLF12 Hs00273134_m1 (NM_007249.4, ex 6-7, 1089, 100 bp); MCM5 Hs01052142_m1 (NM_006739.3, ex 16-17, 2197, 70 bp); and, TFF2 Hs00989207_m1 (NM_005423.4, ex 3-4, 520, 68 bp). For normalization and relative expression assessment, 3 premade TaqMan® MGB assays for endogenous control transcripts were used: #4333767 F for GUSB; Hs00183533_m1 for IPO8; and Hs00427620_m1 for TBP. Samples were run in duplicates, in 10ul reactions (2ul cDNA template per reaction) in an ABI7900HT real time PCR system under default conditions. A commercially available reference RNA derived from multiple transformed cell lines (TaqMan® Control Total RNA, cat. no 4307281, Applied Biosystems) was applied in multiple positions in each run as positive control and for inter-run evaluation of PCR assay efficiency. No-template controls were also included. Samples were run in duplicates, at least in two metachronous runs. To obtain linear Relative Quantification (RQ) values, relative expression was assessed as (40-dCT), as previously described, whereby dCT (or deltaCT) was calculated as (average target CT) – (average endogenous control CT) from all eligible measurements [7]. Samples were considered eligible for analysis when (a) both endogenous control CTs in duplicates were <36 and when duplicate dCT’s for the same sample within the same run were <0.75. The efficiency of all assays was considered as comparable, since the difference between inter-run RQ values for the reference RNA sample was <1 for all assays. Upon testing for target RQ value compliance per sample with each endogenous control, TPB yielded the most unstable results and was thus not included in the final assessment of RQ values.

### Statistical analysis

Analyses of the microarray data were performed using BRB-ArrayTools Software developed by Dr. Richard Simon and BRB-ArrayTools Development Team [8]. After quantile normalization of the samples, we excluded one fourth of the genes showing minimal variation across our dataset. In order to assess gene expression profiles predictive of bevacizumab benefit, we utilized Compound Covariate Predictor, Diagonal Linear Discriminant Analysis, Nearest Neighbor Classification, Support Vector Machines with linear kernel and Bayesian Compound Covariate Predictor. These algorithms incorporate genes differentially expressed among different classes as assessed by the random variance t-test. Evaluation of the predictive value of these methods was based on Leave-One-Out-Cross-Validation. The 8-month Progression-Free status was used as endpoint and surrogate marker of bevacizumab benefit.

For all the markers the median (50th percentile) were examined as possible threshold for prognostic significance categorizing the gene expression levels into high versus low. The expression of five genes was examined for correlation to the following parameters as endpoints: a) Objective response rate (ORR- Best response to therapy; complete or partial response), prior to any metastasectomy, b) Progression-free survival (PFS), calculated from the initiation of first-line therapy to disease progression, death or last follow-up, whichever occurs first.

ORR was chosen as an objective, easy to measure endpoint which is not confounded by the potentially curative resection of metastases in some patients. On the other hand, PFS was used as a survival endpoint in order to investigate the possible association of genes with survival, without impact on tumor regression rates.

The Fisher’s exact test was used to examine possible associations between gene expressions with the overall response rate (ORR), while odds ratios were also calculated in order to measure the association. Time-to-event distributions were estimated using the Kaplan-Meier curves. The log-rank test and Cox’s proportional hazards models was used to examine the univariate prognostic significance of the markers for PFS. For all univariate tests the significance level was set at α = 0.05. Multivariate analysis included clinical parameters and gene expression profiles that were significant in the univariate setting. The SAS software was used for statistical analysis (SAS for Windows, version 9.3, SAS Institute Inc., Cary, NC, USA).

## Results

### Microarray identification of candidate genes in the Test set

**Figure 1:**
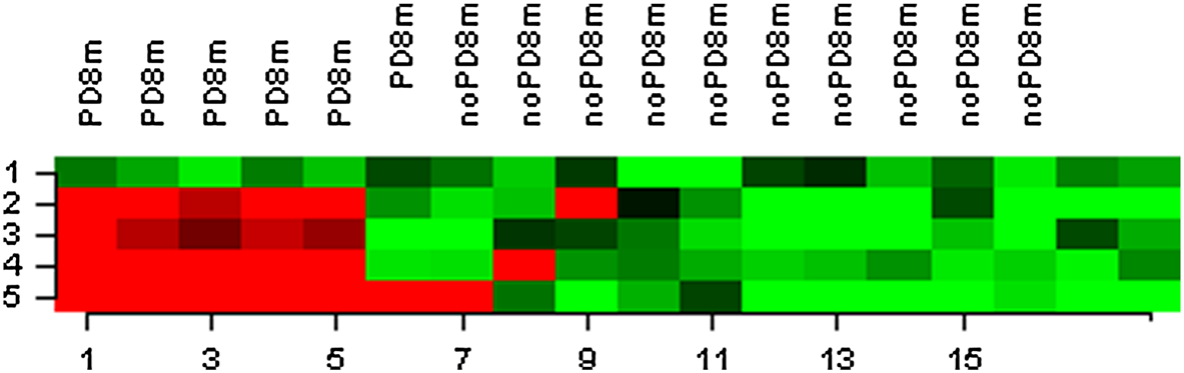
**Gene clustering in microarray analysis of test set according to 8-month progression-free endpoint.** Hierarchical clustering was performed using the euclidean distance and the average linkage algorithm. UPPER ROW-PD8m: Progressive disease during first 8 months from treatment start, noPD8m: Progression-free during 8 months from treatment start. LOWER ROW- Patient tumor samples (n = 16, two patients ineligible). VERTICAL LEFT: Expression of five genes. Red denotes overexpression, Green denotes underexpression.

The sixteen patients in the Test set had metastatic colon cancer treated with FOLFIRI + bevacizumab (n = 13) or CapOx + bevacizumab (n = 3). Three patients had achieved a complete remission and nine a partial remission (objective response rate, ORR 75.0%). Ten patients (62.6%) remained progression-free for at least eight months. Patient and tumor characteristics are shown in Table 1. The 8-month Progression-Free status was used as the endpoint for examining the association of genome-wide gene expression with it as a surrogate marker of bevacizumab activity. In this test set, we developed gene expression models using different algorithms to predict which patients would progress within 8 months. The optimal predictor comprised of five genes, differentially expressed between patients who had progressed within 8 months and those who remained progression free at that time point, at a significance level of 0.00005 (random variance t-test). Prediction accuracies fluctuated between 89–94%, based on the five different algorithms. Tumor tissue samples from patients with unfavorable PFS status were found to overexpress four out of the five genes (AGR2, ALDH6A1, TFF2, MCM5) and underexpress KLF12. Information on the five-gene model can be found in Table 1 and Figure 1.

**Table 1:** Patient demographics and Gene selection in Test set

**Variable**		**Test set**		
***Patients***	***N***	16		
***Age***	***Median***	60.6		
	***Range***	24-71		
		**N (%)**		
***Best response***	***CR***	3 (18.8%)		
	***PR***	9 (56.2%)		
	***SD***	2 (12.6%)		
	***PD***	2 (12.6%)		
***Histological Grade***	***1-2***	10 (62.6%)		
	***3-4***	5 (31.2%)		
***Gender***	***Female***	8 (50%)		
	***Male***	8 (50%)		
***Progressive disease within 8 months***		6 (37.6%)		
***Microarray analysis: Gene selection***				
**Gene**	**Name**	**EntrezID**	**Fold change in 8-month Progression-free cohort**	**FDR**
**KLF12**	**Kruppel-like factor 12**	11278	*1.87*	0.092
**AGR2**	**Anterior gradient homolog 2 (Xenopus laevis)**	10551	*0.62*	0.092
**ALDH6A1**	**Aldehyde dehydrogenase 6 family, member A1**	4329	*0.63*	0.092
**MCM5**	**Minichromosome maintenance complex component 5**	4174	*0.48*	0.157
**TFF2**	**Trefoil factor 2**	7032	*0.45*	0.002

### Application of the 5-gene predictor with qPCR in the Bevacizumab qPCR and Control sets

Application of the 5 genes of the predictor in the Test set yielded significant associations with PFS in the predicted direction for individual markers AGR2, ALDH6A1, MCM5 and TFF2 (favorable PFS for patients with tumors expressing low RQ values, with low at median cut-off) but none for KLF12 (Additional file 2: Figure S2). Predetermined clustering of RQ values for the same genes according to the predictor pattern was not possible for the 16 tumors in this set, since no groups applicable for statistics could be formed. Therefore, the Test set could not be used for the evaluation of the predictor with qPCR.

### Demographics

In the Bevacizumab qPCR set, forty-nine patients with metastatic colon cancer, of a median age of 60.1 years, received mostly irinotecan- or oxaliplatin-based regimens combined with bevacizumab as first-line therapy. An objective response was seen in 36.7% of them, while 51.0% were free from progression for at least 8 months. Seven patients (14.3%) underwent potentially curative metastasectomies following therapy-induced cytoreduction. In the control set, seventy-two advanced colon cancer patients of a median age of 64.5 years received mostly irinotecan-based chemotherapy (58.3%) or oxaliplatin-based regimens as first-line therapy. No bevacizumab was administered. An objective response was seen in 43.1% of them, while 54.2% were free from progression for at least 8 months. Six patients (8.3%) underwent potentially curative metastasectomies following therapy-induced cytoreduction. Most characteristics of the Bevacizumab qPCR and Control sets were matched and are shown in Table 2.

**Table 2:** Patient demographics in Bevacizumab qPCR and Control sets

**Variable**		**Set**		
		**Control**	**Bev qPCR**	**P-value**
***Patients***	***N***	72	49	
***Age***	***Median***	64.5	60.1	0.0543
	***Range***	41-77	32-77	
		**N (%)**	**N (%)**	
***Chemotherapy***				
Irinotecan-based regimen		42 (58.3)	23 (46.9)	0.1084
Oxaliplatin-based regimen		16 (22.2)	21 (42.9)	
Both irinotecan and oxaliplatin		5 (6.9)	1 (2.0)	
Fluoropyrimidine only		2 (2.8)	2 (4.1)	
Erbitux only		7 (9.7)	2 (4.1)	
***Best response***	***CR***	5 (6.9)	1 (2.0)	0.5364
	***PR***	26 (36.1)	17 (34.7)	
	***SD***	16 (22.2)	14 (28.6)	
	***PD***	9 (12.5)	10 (20.4)	
	***NE/Missing Data***	16 (22.3)	7 (14.3)	
***Histological grade***	***1-2***	50 (69.4)	34 (69.4)	0.8638
	***3-4***	19 (26.4)	12 (24.4)	
***Primary site***	***Left***	54 (75.0)	35 (71.4)	0.6619
	***Right***	18 (25.0)	14 (28.6)	
***Gender***	***Female***	24 (33.3)	24 (49.0)	0.0842
	***Male***	48 (66.7)	25 (51.0)	
***Stage at biopsy***	***I-III***	25 (34.8)	16 (32.6)	0.8962
	***IV***	46 (63.8)	31 (63.2)	
***8-month progression-free rate***		39 (54.2)	25 (51.0)	0.6352
***Metastasectomy***		6 (8.3)	7 (14.3)	0.5480

### ORR

**Figure 2:**
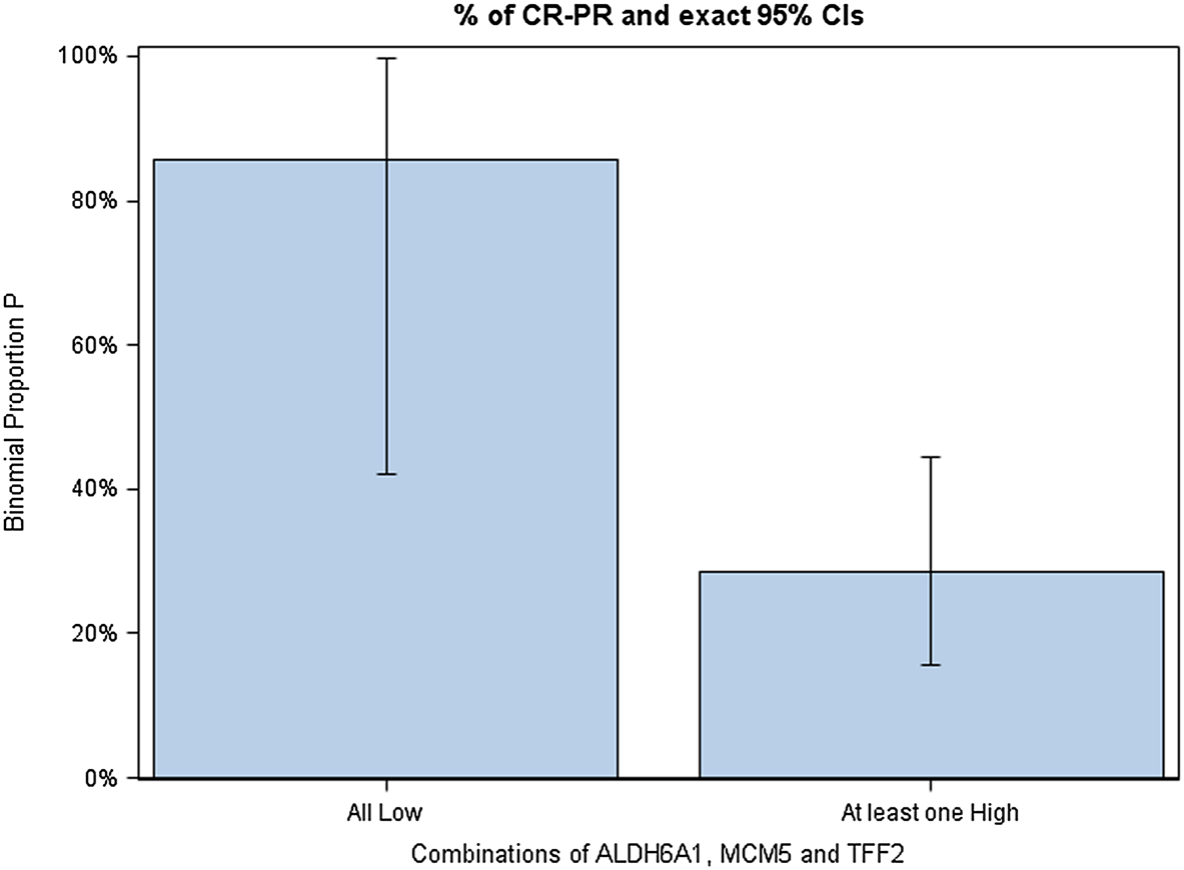
Objective response rate by (ALDH6A1 + TFF2 + MCM5) gene expression in Bevacizumab qPCR set.

The expression of five selected genes individually as well as the complex expression profiles of gene combinations were studied for association with objective response rate. Only the expression profile of ALDH6A1 + TFF2 + MCM5 correlated strongly with response to bevacizumab (see Table 3). Among patients harboring tumors with (ALDH6A1 + TFF2 + MCM5)-all low gene expression, 85.7% responded to bevacizumab chemoimmunotherapy in the Bevacizumab qPCR set versus only 28.6% of those with any other (ALDH6A1 + TFF2 + MCM5) expression profile (p = 0.007). In the Control set, only 36.4% of patients harboring (ALDH6A1 + TFF2 + MCM5)-all low tumors responded to chemotherapy-only, compared to a rather similar ORR of 44.3% in patients with any other (ALDH6A1 + TFF2 + MCM5) expression profile (p = 0.747). The Odds Ratio for Response in patients with (ALDH6A1 + TFF2 + MCM5)-all low tumors compared to any other (ALDH6A1 + TFF2 + MCM5) profile was 0.72 (p = 0.63) in the Control set, but was 15.00 (p = 0.0168) in the Bevacizumab qPCR set. The distribution of patients with tumor regression, stable and progressive disease according to the (ALDH6A1 + TFF2 + MCM5) expression profile is visualized in Figure 2 for the Bevacizumab qPCR set. Of note, low expression of the MCM5 gene also correlated significantly, though less strongly, with ORR (MCM5-low tumors: Bevacizumab qPCR set, ORR 52.0%, p = 0.038 - Control set, ORR 38.9%, p = 0.63).

**Table 3:** Gene expession associated with patient outcomes

	**Control set**	P-value*	**Bev qPCR Set**	P-value*
**ORR**				
***ALDH6A1 + TFF2 + MCM5***				
All low	4/11 (36.4%)	0.747	6/7 (85.7%)	0.007
Other	27/61 (44.3%)		12/42 (28.6%)	
**Odds ratio for response**				
***ALDH6A1 + TFF2 + MCM5***				
All low vs. any other	0.72	0.6273	15.00	0.0168
**Hazard ratio for progression**				
***KLF12 + TFF2***				
Other vs. (KLF12 high + TFF2 low)	1.29	0.39	2.92	0.03
***ALDH6A1 + TFF2***				
Other vs. All low	1.76	0.0404	0.89	0.7406

*Critical point for the significance of p-values is a = 0.05/(N of comparisons) = 0.05/27 = 0.001852 (Bonferroni correction).

### PFS

**Figure 3:**
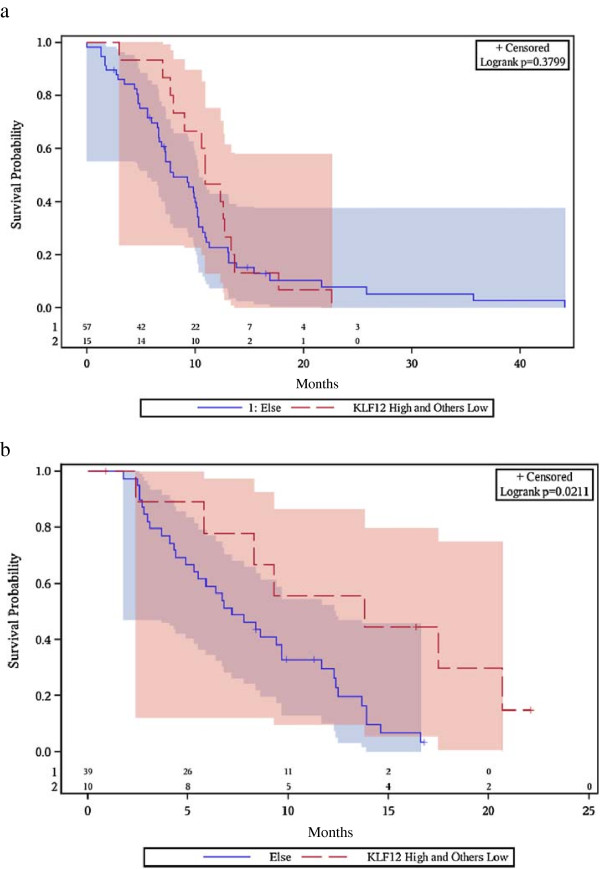
**Progression-free survival by KLF12 + TFF2 gene expression. a)** Control set. **b)** Bevacizumab qPCR set.

When we examined the association of expression of our five genes with PFS, a different picture emerged. In the Bevacizumab qPCR set, patients with (KLF12-high + TFF2-low) tumors had superior PFS (median 14 months, 95% CI 2-21) compared to patients with any other (KLF12 + TFF2) expression profile (median PFS 7 months, 95% CI 5-10, p = 0.021, Figure 3). On the contrary, patients not treated with bevacizumab in the Control set had a median PFS of 11 months (95% CI 8-13) when harboring (KLF12-high + TFF2-low) tumors, not significantly different from the median PFS of 8 months (95% CI 7-10) of patients with any other (KLF12 + TFF2) expression profile (p = 0.38). The Hazard Ratio for risk of progression for any other (KLF12 + TFF2) expression profile versus the reference category of (KLF12-high + TFF2-low) tumors was 2.92 (p = 0.03) in the presence of bevacizumab and 1.29 (p = 0.39) in the absence of bevacizumab.

Of note, the profile of (ALDH6A1 + TFF2 + MCM5) expression did not show any significant association with PFS, while that of (KLF12 + TFF2) no association with tumor response (ORR). Finally, the profile of all-low (ALDH6A1 + TFF2) tumor gene expression showed a marginally significant association with superior survival in the chemotherapy-only Control set, but not in the Bevacizumab qPCR set (Table 3). Detailed information on all examined qPCR gene expression profiles in all sets are given in Additional file 3: Table S1 and Additional file 4: Table S2.

### Multivariate analysis

A multivariate analysis model incorporating performance status, gender, primary site in the colon, type of first line chemotherapy, age, occurrence of metastasectomy and gene expression profiles significant in univariate analyses was applied in the total population of Bevacizumab qPCR and Control sets. No single gene or complex multigene tumor expression profile showed any independent significant association with either ORR or PFS. Parameters with independent prognostic significance for PFS were poor performance status (PS 2-3 vs 0-1, HR for disease progression 18.2, p = 0.0004) and occurrence of metastasectomy (HR for disease progression 0.04, p = 0.0015).

## Discussion

Our microarray-based exploratory study identified the expression of five genes with significant correlation to the probability of disease control from bevacizumab therapy beyond the 8-month benchmark duration. KLF12 (Kruppel-like factor 12) is located at 13q22, encoding a member of the Kruppel-like zinc finger protein family of transcription factors. It represses the expression of the Activator protein-2 alpha (AP-2 alpha) gene, an important regulator of vertebrate development and carcinogenesis, by binding its promoter [9]. As a transcription factor, overexpression of KLF12 in endometrial cancer cell lines significantly repressed proliferation and secretion of pro-survival factors such as insulin-like growth factor binding protein-1 [10]. On the other hand, KLF12 was shown to induce cell proliferation, angiogenesis and invasion in gastric cancer cell lines and clinical samples [11]. High KLF12 gene expession may constitute a surrogate marker of tumors with high proliferative, angiogenic and migratory potential, amenable to VEGF blockade.

TFF2 (Trefoil Factor 2) is located at 21q22.3 and encodes a stable secretory protein, member of the Trefoil family, expressed in gastrointestinal mucosa. Their functions are not defined, but they may protect the mucosa from insults [12]. Breast, pancreas and bile duct cancer cell line experiments suggested that TFF2 expression induces cell migration via Platelet-Activation Receptor 4 (PAR4) and Panc1 activation, as well as mitosis via EGFR/MAPK axis signaling [13-17]. On the other hand, TFF2 was shown to possess anti-inflammatory properties and to undergo promoter methylation during gastric cancer progression, data pointing to a tumor-suppressive function [18,19]. ALDH6A1 (aldehyde dehydrogenase 6 family, member A1) was mapped at 14q24.3. The encoded protein is a mitochondrial methylmalonate semialdehyde dehydrogenase that plays a role in the valine and pyrimidine catabolic pathways. This protein catalyzes the irreversible oxidative decarboxylation of malonate and methylmalonate semialdehydes to acetyl- and propionyl-CoA [20,21]. Despite its regulatory role in mitochondrial energy production and DNA catabolism, no studies examined its putative contribution to cancer homeostasis to date.

MCM5 (minichromosome maintenance complex component 5) at 22q13.1 encodes for a member of the MCM family of chromatin-binding proteins that stimulates cell transition from G0 to G1/S phase of the cell cycle and actively participates in cell cycle regulation [22,23]. Data from clinical and preclinical models of skin, esophageal, bladder and gastrointestinal carcinomas further confirm the proliferative, migratory and cell cycle activating properties of the MCM5 protein [24-27]. Low MCM5 gene expression could mark tumors with low proliferation, abnormal vasculature and hypoxia, the profile most amenable to vessel normalization and cell kill by bevacizumab + chemotherapy. Of note, the most well studied pro-oncogenic gene, AGR2 (Anterior Gradient 2, at 7p21.3) which has identified oncogenic functions such as attenuation of endoplasmic reticulum stress, transition from G0 to G1 phase, inhibition of cell senescence and association with tumor stage, was not found to correlate with either response or progression-free survival in the validation control [28].

We selected a «three-stage» design for our experiment, which should be viewed as hypothesis-generating, rather than proof of principle. First, we used data mining in order to identify genes with potential association with bevacizumab benefit from a test set of 16 patients for whom genome-wide gene expression was studied in a microarray platform. Second, we tested the predictive performance of the 5 genes in the microarray predictor with qPCR, a method more convenient and realistic for clinical practice, in the test set and in an independent Bevacizumab qPCR set of 49 patients who had been treated with bevacizumab. Third, we used the same qPCR approach in order to examine the prognostic significance of the predictor genes in a matched control set of 72 patients who received first-line chemotherapy, but not bevacizumab. We chose to include patients who had metastasectomy after chemotherapy in both cohorts, despite introducing a positive bias: surgical resection of metastases could alter the natural course of disease and be a confounding factor in our search for a predictor of bevacizumab benefit. However, excluding metastasectomy cases would introduce a negative bias, as it is likely that patients led to potentially curative metastasectomy would be the ones with major cytoreduction and disease control from chemotherapy + bevacizumab. Accordingly, we used two metrics for clinical benefit: best tumor response before metastasectomy, which is not confounded by the latter, and progression-free survival, which is more sensitive than overall survival but potentially influenced by metastasectomy. Of note, the incidence of metastasectomy was not significantly different in the Bevacizumab qPCR and Control cohorts. We failed to identify any qPCR gene signature that could incorporate all five genes identified in the Test set, consequently our Bevacizumab qPCR cohort should not be viewed as a «validation» cohort but as an exploratory cohort for the study of a new qPCR signature consisting of some of the preselected five genes.

The conflicting function of genes studied reported by other investigators maybe due to differences in cancer types, tumor microenviroment, expression of multiple other modulating biomolecules, disease stage as well as study design and experimental methodologies. They constitute the interpretation of observed associations of genes with bevacizumab activity extremely difficult, especially in view of lack of constistency when various benefit metrics are examined (response rate, PFS) and the absence of independent significance in multivariate analyses. The inability of our response-predictive qPCR profiles to impact on PFS and vice versa surely raises concerns about the validity of our findings. Still, the decoupling of ORR from PFS benefit may be due to the impact of metastasectomies on PFS [29]. It could also be explained by the commonly reported discrete biologies underlying the phenomena of tumor regression and of control of tumor proliferation, invasion and virulence, especially when anti-angiogenic therapies are administered [30,31]. Technical differences regarding RNA (microarrays) and mRNA (qPCR) sequence detection could also account for the inability to successfully recapitulate the entire 5-gene microarray predictor with qPCR. In view of the multiple analyses performed (see Additional file 3: Table S1 and Additional file 4: Table S2), it is not safe to conclude that the association of our qPCR profiles with clinical benefit from bevacizumab may be reflecting important functional roles of these genes or establish them as surrogate markers of genetic subsets of tumors responsive to anti-angiogenesis. These associations could simply constitute random findings and the data should only be viewed as hypothesis-generating.

Although the search for a validated gene signature predictive for bevacizumab benefit did not bear fruit, some findings are consistently reported. Gene expression profiling studies in bevacizumab-treated patients with glioblastomas, breast and colon cancer identified predictive gene signatures with little or no gene overlap which possessed however a common repertoire of the gene functional ontologies implicated: cell proliferation, mitochondrial energy production, lipid metabolism, migration/invasion, hypoxia regulation and immune response [32-35]. The genes identified here are also characterized by the functional roles above. Brauer et al suggested that a genetic profile predictive for benefit from anti-angiogenesis may be independent of tumor primary, while Fiebig at al could not assign a known function in 59% of the 35 genes predicting for bevacizumab benefit in colorectal cancer xenografts and clinical samples [33,34]. Hu et al reported that although a change in multigene expession in 21 bevacizumab-treated glioblastoma patients correlated to outcome, they could not identify a baseline gene expression signature with prognostic significance. In our case, only gene expression data at baseline were available [36].

## Conclusions

To conclude, we identified two distinct qPCR gene expression profiles correlating with response (low expression of ALDH6A1 + TFF2 + MCM5) and with PFS (KLF12-high + TFF2-low) in advanced colon cancer patients managed with bevacizumab and chemotherapy. Despite our three-cohort experimental design, the moderate sample size, the plethora of variables under study and of analyses performed preclude us from establishing a predictive utillity for these genetic profiles before further validation in independent cohorts.

## Competing interests

The authors declare that they have no competing interests.

## Authors’ contribution

GP contributed to conception and coordination of the study, analysis and interpretation of data, writing of the manuscript. VK contributed to design of RT-PCR experiments, conception of the study, analysis and interpretation of data. EF contributed to design of microarray experiments, analysis and interpretation of data. GK contributed to to analysis and interpretation of data. GF contributed to conception of the study, analysis and interpretation of data. All other authors contributed to analysis and interpretation of data. All authors read and approved the final manuscript.

## Pre-publication history

The pre-publication history for this paper can be accessed here:

http://www.biomedcentral.com/1471-2407/14/111/prepub

## Supplementary Material

Additional file 1: Figure S1 REMARK diagram. Click here for file

Additional file 2: Figure S2 Performance of all 5 genes of the microarray predictor in the test, validation and control sets with respect to patient PFS. AGR2, ALDH6A1 and MCM2 were consistent with the predictor in the test set but not in the validation set; instead, 2 out of 3 genes were associated with longer PFS in the non-bevacizumab treated cohort. TFF2 was consistent with the predictor in the test and validation sets. KLF12 was the only gene that could not be validated in the test set with qPCR; high transcript levels of this gene were, however, showed a trend for better outcome in the validation set. Based on these findings and upon failure to transfer the entire 5-gene signature into a single qPCR profile, KLF12 and TFF2 RQ values were profiled for assessing their possible value in predicting PFS upon bevacizumab treatment. Click here for file

Additional file 3: Table S1 Univariate Cox regression for each qPCR gene expression and their combinations among dataset groups in terms of PFS. Click here for file

Additional file 4: Table S2 Fisher’s exact test for each qPCR gene expression and their combinations among dataset groups in terms of ORR. Click here for file
