# Supplementary material for: A study of gene expression markers for predictive significance for bevacizumab benefit in patients with metastatic colon cancer: a translational research study of the Hellenic Cooperative Oncology Group (HeCOG)
Source: BMC Cancer. 2014 Feb 20;14:111. doi: 10.1186/1471-2407-14-111 (PMC3933361; doi:10.1186/1471-2407-14-111)
Supplement: Additional file 1: Figure S1 — REMARK diagram. [file 1471-2407-14-111-S1.doc]

REMARK diagram.

FFPE blocks retrospectively identified

Test Set

(n = 18)

Validation Set

(n=62)

Control Set

(n=82)

**Block collection**

Excluded/Not recovered (n = 12)

Not meeting quality microarray criteria

(n = 2, Test Set)

Lack of clinicopathologic data

(Validation set n=4, Control Set n=6)

Test Set

(n = 16)

Validation Set

(n=58)

Control Set

(n=76)

Excluded/Not recovered (n = 13)

Poor tumour cellularity

(Validation Set n = 5)

Poor quality/quantity of extracted RNA

(Validation set n=4, Control Set n=4)

#

**Successful H &E evaluation, nucleic acid extraction and quality control**

Analysed

Test Set

(n = 16)

Validation Set

(n=49)

Control Set

(n=72)
