# Supplementary figures and images for: A study of gene expression markers for predictive significance for bevacizumab benefit in patients with metastatic colon cancer: a translational research study of the Hellenic Cooperative Oncology Group (HeCOG)
Source: BMC Cancer. 2014 Feb 20;14:111. doi: 10.1186/1471-2407-14-111 (PMC3933361; doi:10.1186/1471-2407-14-111)

## Slide 1
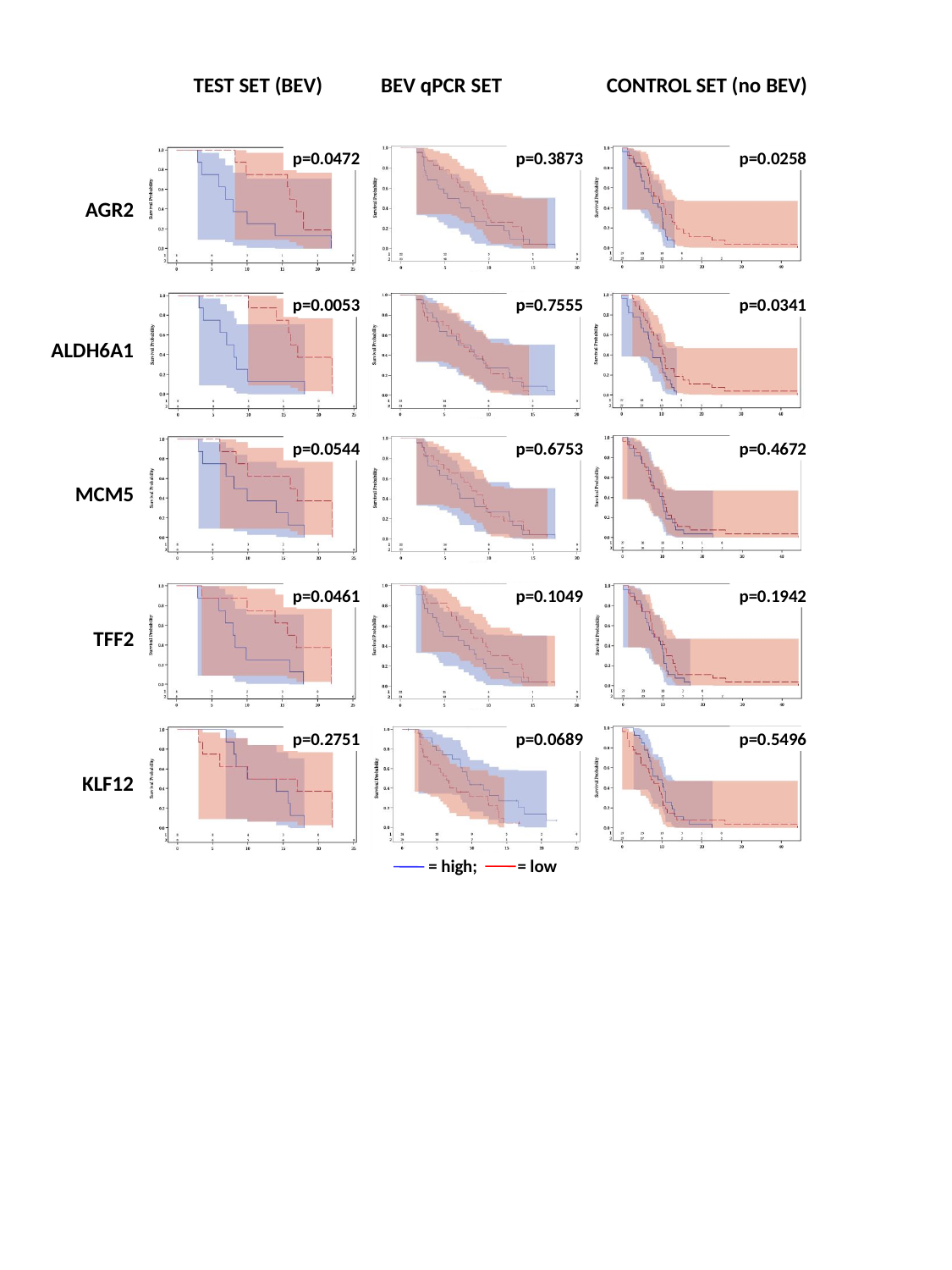

TEST SET (BEV)
BEV qPCR SET
CONTROL SET (no BEV)
p=0.0472
p=0.3873
p=0.0258
AGR2
p=0.0053
p=0.7555
p=0.0341
ALDH6A1
p=0.0544
p=0.6753
p=0.4672
MCM5
p=0.0461
p=0.1049
p=0.1942
TFF2
p=0.2751
p=0.0689
p=0.5496
KLF12
= high; = low

Supplement: Additional file 2: Figure S2 — Performance of all 5 genes of the microarray predictor in the test, validation and control sets with respect to patient PFS. AGR2, ALDH6A1 and MCM2 were consistent with the predictor in the test set but not in the validation set; instead, 2 out of 3 genes were associated with longer PFS in the non-bevacizumab treated cohort. TFF2 was consistent with the predictor in the test and validation sets. KLF12 was the only gene that could not be validated in the test set with qPCR; high transcript levels of this gene were, however, showed a trend for better outcome in the validation set. Based on these findings and upon failure to transfer the entire 5-gene signature into a single qPCR profile, KLF12 and TFF2 RQ values were profiled for assessing their possible value in predicting PFS upon bevacizumab treatment. [file 1471-2407-14-111-S2.ppt]
