# Supplementary material for: A study of gene expression markers for predictive significance for bevacizumab benefit in patients with metastatic colon cancer: a translational research study of the Hellenic Cooperative Oncology Group (HeCOG)
Source: BMC Cancer. 2014 Feb 20;14:111. doi: 10.1186/1471-2407-14-111 (PMC3933361; doi:10.1186/1471-2407-14-111)
Supplement: Additional file 3: Table S1 — Univariate Cox regression for each qPCR gene expression and their combinations among dataset groups in terms of PFS. [file 1471-2407-14-111-S3.doc]

**Supplementary Table 1:** Univariate Cox regression for each qPCR gene expression and their combinations among dataset groups in terms of PFS.

|  |  | **Dataset** | | | | | |
| --- | --- | --- | --- | --- | --- | --- | --- |
|  |  | **Test** | | **Bevacizumab qPCR** | | **Control** | |
| **Parameter** |  | **Hazard Ratio** | **P-value*** | **Hazard Ratio** | **P-value*** | **Hazard Ratio** | **P-value*** |
|  |  |  |  |  |  |  |  |
| AGR2 gusb/ipo8 | Low vs. High | 2.91 | 0.06 | 1.12 | 0.71 | 1.34 | 0.24 |
| ALDH6A1 gusb/ipo8 | Low vs. High | 4.75 | 0.01 | 0.68 | 0.23 | 1.32 | 0.27 |
| KLF12 gusb/ipo8 | Low vs. High | 1.88 | 0.28 | 0.56 | 0.07 | 0.94 | 0.80 |
| MCM5 gusb/ipo8 | Low vs. High | 2.94 | 0.07 | 0.70 | 0.27 | 1.33 | 0.25 |
| TFF2 gusb/ipo8 | Low vs. High | 3.05 | 0.06 | 1.65 | 0.13 | 1.36 | 0.22 |
| Combination of All 4 genes | All low vs. At least one high | 7.27 | 0.06 | 0.91 | 0.84 | 1.66 | 0.19 |
| AGR2 and ALDH6A1 | All low vs. At least one high | 3.70 | 0.05 | 0.90 | 0.77 | 1.41 | 0.21 |
| AGR2 and MCM5 | All low vs. At least one high | 3.12 | 0.09 | 0.89 | 0.75 | 1.37 | 0.26 |
| AGR2 and TFF2 | All low vs. At least one high | 4.32 | 0.06 | 1.13 | 0.71 | 1.57 | 0.10 |
| ALDH6A1 and MCM5 | All low vs. At least one high | 5.46 | 0.01 | 0.73 | 0.33 | 1.10 | 0.71 |
| ALDH6A1 and TFF2 | All low vs. At least one high | 4.23 | 0.02 | 0.89 | 0.74 | 1.76 | 0.04 |
| MCM5 and TFF2 | All low vs. At least one high | 6.36 | 0.02 | 1.02 | 0.96 | 1.30 | 0.37 |
| AGR2, ALDH6A1 and MCM5 | All low vs. At least one high | 4.66 | 0.05 | 1.04 | 0.94 | 1.31 | 0.40 |
| AGR2, ALDH6A1 and TFF2 | All low vs. At least one high | 4.32 | 0.06 | 0.82 | 0.60 | 1.75 | 0.09 |
| AGR2, MCM5 and TFF2 | All low vs. At least one high | 7.27 | 0.06 | 0.77 | 0.53 | 1.60 | 0.16 |
| ALDH6A1, MCM5 and TFF2 | All low vs. At least one high | 6.36 | 0.02 | 1.00 | 1.00 | 1.50 | 0.25 |
| KLF12 and AGR2 | KLF12 high and others low vs. Else | 0.89 | 0.84 | 1.58 | 0.22 | 1.39 | 0.26 |
| KLF12 and ALDH6A1 | KLF12 high and others low vs. Else | 0.92 | 0.90 | 0.91 | 0.82 | 1.15 | 0.64 |
| KLF12 and MCM5 | KLF12 high and others low vs. Else | 0.74 | 0.70 | 0.83 | 0.62 | 1.22 | 0.55 |
| KLF12 and TFF2 | KLF12 high and others low vs. Else | 0.83 | 0.81 | 2.92 | 0.03 | 1.29 | 0.39 |
| KLF12, AGR2 and ALDH6A1 | KLF12 high and others low vs. Else | 1.02 | 0.98 | 0.99 | 0.99 | 1.56 | 0.22 |
| KLF12, AGR2 and MCM5 | KLF12 high and others low vs. Else | 0.74 | 0.70 | 1.02 | 0.97 | 1.44 | 0.43 |
| KLF12, AGR2 and TFF2 | KLF12 high and others low vs. Else | 0.92 | 0.94 | 1.84 | 0.21 | 1.52 | 0.23 |
| KLF12, ALDH6A1 and MCM5 | KLF12 high and others low vs. Else | 1.11 | 0.92 | 0.94 | 0.90 | 0.95 | 0.91 |
| KLF12, ALDH6A1 and TFF2 | KLF12 high and others low vs. Else | 0.83 | 0.81 | 0.83 | 0.73 | 1.44 | 0.34 |
| KLF12, AGR2, ALDH6A1 and MCM5 | KLF12 high and others low vs. Else | 1.11 | 0.92 | 1.21 | 0.79 | 1.31 | 0.60 |
| KLF12, AGR2, ALDH6A1 and TFF2 | KLF12 high and others low vs. Else | 0.92 | 0.94 | 0.83 | 0.73 | 1.66 | 0.28 |

*Critical point for the significance of p-values is a=0.05/(N of comparisons)=0.05/27=0.001852 (Bonferroni correction)
