# Supplementary material for: A study of gene expression markers for predictive significance for bevacizumab benefit in patients with metastatic colon cancer: a translational research study of the Hellenic Cooperative Oncology Group (HeCOG)
Source: BMC Cancer. 2014 Feb 20;14:111. doi: 10.1186/1471-2407-14-111 (PMC3933361; doi:10.1186/1471-2407-14-111)
Supplement: Additional file 4: Table S2 — Fisher’s exact test for each qPCR gene expression and their combinations among dataset groups in terms of ORR. [file 1471-2407-14-111-S4.doc]

**Supplementary Table 2:** Fisher’s exact test for each qPCR gene expression and their combinations among dataset groups in terms of ORR.

|  |  | **Dataset** | | | | | | | | | | | |
| --- | --- | --- | --- | --- | --- | --- | --- | --- | --- | --- | --- | --- | --- |
|  |  | **Test** | | | | **Bevacizumab qPCR** | | | | **Control** | | | |
| **Parameter** |  | **CR-PR** | **Other** | **Odds Ratio** | **P-value*** | **CR-PR** | **Other** | **Odds Ratio** | **P-value*** | **CR-PR** | **Other** | **Odds Ratio** | **P-value*** |
|  |  |  |  |  |  |  |  |  |  |  |  |  |  |
| AGR2 gusb/ipo8 cut off at | High | 4 (50.0) | 4 (50.0) |  | 0.077 | 7 (29.2) | 17 (70.8) | 0,52 | 0.377 | 16 (44.4) | 20 (55.6) | 1,12 | 1.000 |
| median | Low | 8 (100.0) |  |  |  | 11 (44.0) | 14 (56.0) |  |  | 15 (41.7) | 21 (58.3) |  |  |
|  |  |  |  |  |  |  |  |  |  |  |  |  |  |
| ALDH6A1 gusb/ipo8 cut off at | High | 4 (50.0) | 4 (50.0) |  | 0.077 | 8 (33.3) | 16 (66.7) | 0.75 | 0.769 | 16 (44.4) | 20 (55.6) | 1.12 | 1.000 |
| median | Low | 8 (100.0) |  |  |  | 10 (40.0) | 15 (60.0) |  |  | 15 (41.7) | 21 (58.3) |  |  |
|  |  |  |  |  |  |  |  |  |  |  |  |  |  |
| KLF12 gusb/ipo8 cut off at | High | 7 (87.5) | 1 (12.5) | 4.20 | 0.569 | 9 (37.5) | 15 (62.5) | 1.07 | 1.000 | 16 (44.4) | 20 (55.6) | 1.12 | 1.000 |
| median | Low | 5 (62.5) | 3 (37.5) |  |  | 9 (36.0) | 16 (64.0) |  |  | 15 (41.7) | 21 (58.3) |  |  |
|  |  |  |  |  |  |  |  |  |  |  |  |  |  |
| MCM5 gusb/ipo8 cut off at | High | 5 (62.5) | 3 (37.5) | 0.24 | 0.569 | 5 (20.8) | 19 (79.2) | 0.24 | **0.038** | 17 (47.2) | 19 (52.8) | 1.41 | 0.634 |
| median | Low | 7 (87.5) | 1 (12.5) |  |  | 13 (52.0) | 12 (48.0) |  |  | 14 (38.9) | 22 (61.1) |  |  |
|  |  |  |  |  |  |  |  |  |  |  |  |  |  |
| TFF2 gusb/ipo8 cut off at | High | 5 (62.5) | 3 (37.5) | 0.24 | 0.569 | 8 (33.3) | 16 (66.7) | 0.75 | 0.769 | 14 (38.9) | 22 (61.1) | 0.71 | 0.634 |
| median | Low | 7 (87.5) | 1 (12.5) |  |  | 10 (40.0) | 15 (60.0) |  |  | 17 (47.2) | 19 (52.8) |  |  |
|  |  |  |  |  |  |  |  |  |  |  |  |  |  |
| Combinaltion of All 4 genes | All low | 3 (100.0) |  |  | 0.529 | 4 (80.0) | 1 (20.0) | 8.57 | 0.054 | 3 (33.3) | 6 (66.7) | 0.63 | 0.723 |
|  | At least one high | 9 (69.2) | 4 (30.8) |  |  | 14 (31.8) | 30 (68.2) |  |  | 28 (44.4) | 35 (55.6) |  |  |
|  |  |  |  |  |  |  |  |  |  |  |  |  |  |
| Combinations of AGR2 and | All low | 5 (100.0) |  |  | 0.245 | 6 (46.2) | 7 (53.8) | 1.71 | 0.508 | 9 (42.9) | 12 (57.1) | 0.99 | 1.000 |
| ALDH6A1 | At least one high | 7 (63.6) | 4 (36.4) |  |  | 12 (33.3) | 24 (66.7) |  |  | 22 (43.1) | 29 (56.9) |  |  |
|  |  |  |  |  |  |  |  |  |  |  |  |  |  |
| Combinations of AGR2 and | All low | 5 (100.0) |  |  | 0.245 | 9 (69.2) | 4 (30.8) | 6.75 | **0.007** | 7 (33.3) | 14 (66.7) | 0.56 | 0.310 |
| MCM5 | At least one high | 7 (63.6) | 4 (36.4) |  |  | 9 (25.0) | 27 (75.0) |  |  | 24 (47.1) | 27 (52.9) |  |  |
|  |  |  |  |  |  |  |  |  |  |  |  |  |  |
| Combinations of AGR2 and | All low | 4 (100.0) |  |  | 0.516 | 6 (40.0) | 9 (60.0) | 1.22 | 0.759 | 10 (45.5) | 12 (54.5) | 1.15 | 0.802 |
| TFF2 | At least one high | 8 (66.7) | 4 (33.3) |  |  | 12 (35.3) | 22 (64.7) |  |  | 21 (42.0) | 29 (58.0) |  |  |
|  |  |  |  |  |  |  |  |  |  |  |  |  |  |
| Combinations of ALDH6A1 and | All low | 6 (100.0) |  |  | 0.234 | 9 (56.3) | 7 (43.8) | 3.43 | 0.063 | 7 (33.3) | 14 (66.7) | 0.56 | 0.310 |
| MCM5 | At least one high | 6 (60.0) | 4 (40.0) |  |  | 9 (27.3) | 24 (72.7) |  |  | 24 (47.1) | 27 (52.9) |  |  |
|  |  |  |  |  |  |  |  |  |  |  |  |  |  |
| Combinations of ALDH6A1 and | All Low | 7 (100.0) |  |  | 0.088 | 6 (50.0) | 6 (50.0) | 2.08 | 0.316 | 9 (42.9) | 12 (57.1) | 0.99 | 1.000 |
| TFF2 | At least one High | 5 (55.6) | 4 (44.4) |  |  | 12 (32.4) | 25 (67.6) |  |  | 22 (43.1) | 29 (56.9) |  |  |
|  |  |  |  |  |  |  |  |  |  |  |  |  |  |
| Combinations of MCM5 and | All low | 5 (100.0) |  |  | 0.245 | 7 (63.6) | 4 (36.4) | 4.30 | 0.072 | 9 (50.0) | 9 (50.0) | 1.45 | 0.586 |
| TFF2 | At least one high | 7 (63.6) | 4 (36.4) |  |  | 11 (28.9) | 27 (71.1) |  |  | 22 (40.7) | 32 (59.3) |  |  |
|  |  |  |  |  |  |  |  |  |  |  |  |  |  |
| Combinations of AGR2, | All low | 4 (100.0) |  |  | 0.516 | 5 (71.4) | 2 (28.6) | 5.58 | 0.084 | 4 (28.6) | 10 (71.4) | 0.46 | 0.249 |
| ALDH6A1 and MCM5 | At least one high | 8 (66.7) | 4 (33.3) |  |  | 13 (31.0) | 29 (69.0) |  |  | 27 (46.6) | 31 (53.4) |  |  |
|  |  |  |  |  |  |  |  |  |  |  |  |  |  |
| Combinations of AGR2, | All low | 4 (100.0) |  |  | 0.516 | 4 (44.4) | 5 (55.6) | 1.49 | 0.708 | 6 (46.2) | 7 (53.8) | 1.17 | 1.000 |
| ALDH6A1 and TFF2 | At least one high | 8 (66.7) | 4 (33.3) |  |  | 14 (35.0) | 26 (65.0) |  |  | 25 (42.4) | 34 (57.6) |  |  |
|  |  |  |  |  |  |  |  |  |  |  |  |  |  |
| Combinations of AGR2, MCM5 | All low | 3 (100.0) |  |  | 0.529 | 5 (71.4) | 2 (28.6) | 5.58 | 0.084 | 5 (38.5) | 8 (61.5) | 0.79 | 0.767 |
| and TFF2 | At least one high | 9 (69.2) | 4 (30.8) |  |  | 13 (31.0) | 29 (69.0) |  |  | 26 (44.1) | 33 (55.9) |  |  |
|  |  |  |  |  |  |  |  |  |  |  |  |  |  |
| Combinations of ALDH6A1, | All low | 5 (100.0) |  |  | 0.245 | 6 (85.7) | 1 (14.3) | 15.00 | **0.007** | 4 (36.4) | 7 (63.6) | 0.72 | 0.747 |
| MCM5 and TFF2 | At least one high | 7 (63.6) | 4 (36.4) |  |  | 12 (28.6) | 30 (71.4) |  |  | 27 (44.3) | 34 (55.7) |  |  |
|  |  |  |  |  |  |  |  |  |  |  |  |  |  |
| Combinations of KLF12 and AGR2 | Else | 7 (63.6) | 4 (36.4) |  | 0.245 | 12 (33.3) | 24 (66.7) | 0.58 | 0.508 | 24 (44.4) | 30 (55.6) | 1.26 | 0.787 |
|  | KLF12 high and others low | 5 (100.0) |  |  |  | 6 (46.2) | 7 (53.8) |  |  | 7 (38.9) | 11 (61.1) |  |  |
|  |  |  |  |  |  |  |  |  |  |  |  |  |  |
| Combinations of KLF12 and | Else | 9 (69.2) | 4 (30.8) |  | 0.529 | 15 (40.5) | 22 (59.5) | 2.05 | 0.494 | 23 (41.1) | 33 (58.9) | 0.70 | 0.576 |
| ALDH6A1 | KLF12 high and others low | 3 (100.0) |  |  |  | 3 (25.0) | 9 (75.0) |  |  | 8 (50.0) | 8 (50.0) |  |  |
|  |  |  |  |  |  |  |  |  |  |  |  |  |  |
| Combinations of KLF12 and MCM5 | Else | 10 (71.4) | 4 (28.6) |  | 1.000 | 12 (31.6) | 26 (68.4) | 0.38 | 0.286 | 27 (45.0) | 33 (55.0) | 1.64 | 0.536 |
|  | KLF12 high and others low | 2 (100.0) |  |  |  | 6 (54.5) | 5 (45.5) |  |  | 4 (33.3) | 8 (66.7) |  |  |
|  |  |  |  |  |  |  |  |  |  |  |  |  |  |
| Combinations of KLF12 and TFF2 | Else | 10 (71.4) | 4 (28.6) |  | 1.000 | 15 (38.5) | 24 (61.5) | 1.46 | 0.726 | 22 (38.6) | 35 (61.4) | 0.42 | 0.155 |
|  | KLF12 high and others low | 2 (100.0) |  |  |  | 3 (30.0) | 7 (70.0) |  |  | 9 (60.0) | 6 (40.0) |  |  |
|  |  |  |  |  |  |  |  |  |  |  |  |  |  |
| Combinations of KLF12, AGR2 | Else | 10 (71.4) | 4 (28.6) |  | 1.000 | 16 (37.2) | 27 (62.8) | 1.19 | 1.000 | 27 (42.9) | 36 (57.1) | 0.94 | 1.000 |
| and ALDH6A1 | KLF12 high and others low | 2 (100.0) |  |  |  | 2 (33.3) | 4 (66.7) |  |  | 4 (44.4) | 5 (55.6) |  |  |
|  |  |  |  |  |  |  |  |  |  |  |  |  |  |
| Combinations of KLF12, AGR2 | Else | 10 (71.4) | 4 (28.6) |  | 1.000 | 13 (31.0) | 29 (69.0) | 0.18 | 0.084 | 30 (45.5) | 36 (54.5) | 4.17 | 0.227 |
| and MCM5 | KLF12 high and others low | 2 (100.0) |  |  |  | 5 (71.4) | 2 (28.6) |  |  | 1 (16.7) | 5 (83.3) |  |  |
|  |  |  |  |  |  |  |  |  |  |  |  |  |  |
| Combinations of KLF12, AGR2 | Else | 11 (73.3) | 4 (26.7) |  | 1.000 | 16 (38.1) | 26 (61.9) | 1.54 | 1.000 | 26 (41.9) | 36 (58.1) | 0.72 | 0.736 |
| and TFF2 | KLF12 high and others low | 1 (100.0) |  |  |  | 2 (28.6) | 5 (71.4) |  |  | 5 (50.0) | 5 (50.0) |  |  |
|  |  |  |  |  |  |  |  |  |  |  |  |  |  |
| Combinations of KLF12, | Else | 11 (73.3) | 4 (26.7) |  | 1.000 | 15 (34.9) | 28 (65.1) | 0.54 | 0.656 | 28 (43.1) | 37 (56.9) | 1.01 | 1.000 |
| ALDH6A1 and MCM5 | KLF12 high and others low | 1 (100.0) |  |  |  | 3 (50.0) | 3 (50.0) |  |  | 3 (42.9) | 4 (57.1) |  |  |
|  |  |  |  |  |  |  |  |  |  |  |  |  |  |
| Combinations of KLF12, | Else | 10 (71.4) | 4 (28.6) |  | 1.000 | 17 (38.6) | 27 (61.4) | 2.52 | 0.639 | 26 (40.6) | 38 (59.4) | 0.41 | 0.278 |
| ALDH6A1 and TFF2 | KLF12 high and others low | 2 (100.0) |  |  |  | 1 (20.0) | 4 (80.0) |  |  | 5 (62.5) | 3 (37.5) |  |  |
|  |  |  |  |  |  |  |  |  |  |  |  |  |  |
| Combinations of KLF12, AGR2, | Else | 11 (73.3) | 4 (26.7) |  | 1.000 | 16 (34.8) | 30 (65.2) | 0.27 | 0.546 | 30 (44.1) | 38 (55.9) | 2.37 | 0.629 |
| ALDH6A1 and MCM5 | KLF12 high and others low | 1 (100.0) |  |  |  | 2 (66.7) | 1 (33.3) |  |  | 1 (25.0) | 3 (75.0) |  |  |
|  |  |  |  |  |  |  |  |  |  |  |  |  |  |
| Combinations of KLF12, AGR2, | Else | 11 (73.3) | 4 (26.7) |  | 1.000 | 17 (37.8) | 28 (62.2) | 1.82 | 1.000 | 28 (41.8) | 39 (58.2) | 0.48 | 0.646 |
| ALDH6A1 and TFF2 | KLF12 high and others low | 1 (100.0) |  |  |  | 1 (25.0) | 3 (75.0) |  |  | 3 (60.0) | 2 (40.0) |  |  |

*Critical point for the significance of p-values is a=0.05/(N of comparisons)=0.05/27=0.001852 (Bonferroni correction)
